# Layered double hydroxide materials for environmental applications: insights on key properties from synthesis and structure

**DOI:** 10.1039/d6ra02483a

**Published:** 2026-04-28

**Authors:** Zineb Bouziane, Fouad Amor, Sara Fatine, Jean-Michel Nunzi, Abdelaziz Laghzizil

**Affiliations:** a Laboratory of Applied Chemistry of Materials, Faculty of Sciences, Mohammed V University in Rabat Avenue Ibn Batouta BP.1014 Agdal Rabat Morocco; b Department of Physics, Engineering Physics and Astronomy, Department of Chemistry, Queen's University Kingston ON K7L 3N6 Canada nunzijm@queensu.ca

## Abstract

Layered double hydroxides (LDHs) are layered materials of increasing interest for environmental applications due to their tunable chemical composition, structure, and adjustable physicochemical properties. This review presents a critical synthesis of recent advances in LDH-based materials, highlighting the close links between synthesis methods, structural characteristics, and key properties controlling their environmental performance. The main synthesis strategies are discussed in relation to their influence on crystallinity, morphology, specific surface area, metal cation distribution, and the nature of structural defects. Particular attention is paid to the effect of cationic composition, interlayer anions, and structural modifications (doping, exfoliation, composite formation) on adsorption, ion exchange, redox activity, and heterogeneous photocatalysis mechanisms. Environmental applications of LDHs are systematically examined, including the adsorption of inorganic and organic pollutants, the photodegradation of emerging contaminants under UV and visible irradiation, and water treatment. LDH-derived materials, particularly mixed metal oxides and LDH/semiconductor composites, are also discussed due to their improved photocatalytic performance and increased stability. Finally, current challenges and future prospects are addressed, with a particular focus on the recyclability, durability, and scaling up of LDH-based materials for advanced environmental applications.

## Abstract 2

Relationships between structural features, chemical composition, and environmental applications of Layered Double Hydroxides (LDHs) are critically reviewed.

## Graphical abstract

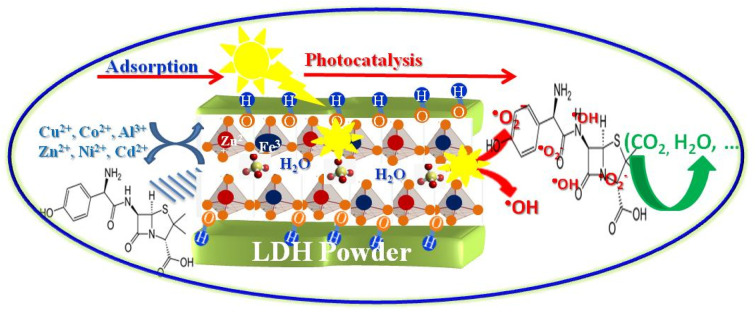

## 1. Introduction

Lamellar structures are interesting advanced functional materials due to their anisotropy, large specific surface area, and high capacity for chemical modulation. Among them, layered double hydroxides (LDH) are characterized by a unique architecture derived from brucite Mg(OH)_2_, in which a fraction of the divalent cations are substituted by trivalent cations, inducing a positive charge in the layers, compensated by hydrated interlayer anions. Their general formula M^II^_1−*x*_M^III^_*x*_(OH)_2_]^*x*+^(A^*n*−^)_*x*/*n*_, mH_2_O reflects their remarkable chemical flexibility, which can incorporate a wide variety of metallic cations and inorganic or organic anions, thereby endowing LDHs with adjustable physicochemical properties.^1–3^ Thanks to the synergy between metal cations and the abundance of redox sites, LDHs are widely employed in electrocatalysis, energy storage, and advanced electrochemical devices.^4,5^ Biocompatibility and intercalation capacity also make these materials attractive for controlled drug delivery and biomedical applications.^6^ Furthermore, LDHs are used in intelligent corrosion protection systems, providing active protection through anion exchange and the controlled release of inhibitors. Recently, research interest in these materials has intensified significantly owing to their strong potential in environmental applications.^7,8^ This rising attention stems from the ability to precisely adjust their chemical composition, layered structure, morphology, and surface characteristics *via* customized synthesis methods.^9^ Synthetic methods play a crucial role in the structuring of LDHs. Coprecipitation and hydrothermal methods are the most commonly used due to their simplicity and low cost. However, alternative advanced techniques, including microwave or ultrasound-assisted syntheses, as well as mechanochemical and electrochemical methods are also under investigation.^10–12^ they have demonstrated their effectiveness in improving crystallinity, modulating layer size, and inducing specific hierarchical morphologies. Furthermore, the synthesis temperature, aging time, pH of the reaction medium, and the M^2+^/M^3+^ molar ratio directly influence the layer stacking, the charge density of the layers, and the nature of the intercalated anions. Beyond conventional commercial precursors, recent studies have increasingly emphasized the synthesis of LDHs from natural resources or industrial waste, aiming at sustainable chemistry and circular economy principles.^13,14^ Indeed, employing natural minerals rich in Mg, Al, Fe, or Ca, as well as industrial sludge or mining residues, not only lowers production costs but also valorizes waste.^15^ yielding materials with properties comparable to or even surpassing those of LDHs synthesized from pure salts.^16^ These approaches nevertheless raise challenges related to purity, reproducibility, and control of the final chemical composition.

LDH materials have attracted increasing interest in environmental applications thanks to their exceptional combination of structural, textural, and chemical properties.^17^ They have demonstrated significant effectiveness in removing emerging polluting species, while also establishing themselves as promising platforms in adsorption, catalysis, and photocatalysis processes.^7,18,19^ In wastewater treatment, LDHs have demonstrated a remarkable capacity for adsorbing and removing heavy metals through mechanisms combining complexation, precipitation, ion exchange, and electrostatic interactions.^20–22^ For example, functionalized LDHs have been successfully used to remove ions such as Pb^2+^, Cd^2+^, Cr^6+^, or Cu^2+^ from aqueous solutions, taking advantage of their large active surface area and available chemical sites for selective adsorption.^23^ Beyond heavy metals, LDHs are effective at capturing toxic oxyanions such as phosphates and nitrates, which are responsible for eutrophication in aquatic systems.^24^ In photocatalysis, LDHs are particularly attractive due to their tunable semiconducting properties, their ability to absorb light in the UV-visible spectrum, and their capacity to promote the separation of photogenerated electron–hole pairs,^17,25^ This makes them suitable for applications such as the degradation of organic pollutants,^19,26,27^ and the photocatalytic reduction of CO_2_.^28^ Modifying LDHs *via* doping, heterojunctions, defects, or conductive supports enhances light absorption, charge separation, and photocatalytic performance.^7,29,30^ In this context, this critical and comprehensive overview of layered double hydroxides (LDHs) provides a guide for readers to familiarize with their fundamental principles and environmental relevance. It focuses on LDH synthesis strategies using both commercial and natural precursors, and systematically discusses how synthesis parameters, chemical composition, and thermal treatments influence their structural and physicochemical properties. The review further establishes clear relationships between structure, properties, and performance, with particular emphasis on adsorption and photocatalytic applications for environmental remediation. By highlighting current environmental challenges and recent advances, this work clarifies the key factors governing LDH efficiency and supports the rational design of high-performance and sustainable LDH-based materials for future industrial applications.

## 2. Multifunctional applications of LDH materials

**Fig. 1:**
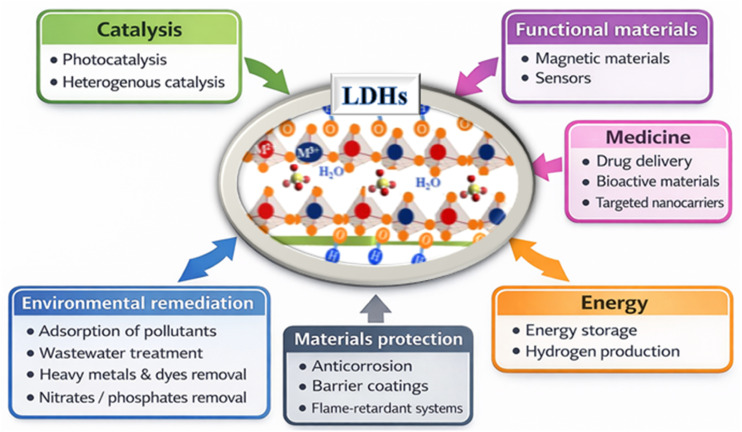
Schematic representation of the diverse applications of LDH based materials.

The unique architecture of LDHs, with positively charged brucite-like layers composed of divalent (M^2+^) and trivalent (M^3+^) metal cations and charge-compensating anions and water molecules located in the interlayer spaces, allows for precise control of composition, morphology. That functionality makes LDHs attractive for a wide range of applications (Fig. 1). One of the most studied applications of LDHs (low-density hydrogen peroxide) is in environmental remediation, particularly water treatment and purification.^31^ Their positively charged layers and the presence of exchangeable interlayer anions give them excellent adsorption and ion exchange capacities. They are highly effective at removing anionic contaminants such as nitrates, phosphates, chromates, and organic dyes from aqueous solutions. Furthermore, LDHs can immobilize or adsorb toxic heavy metal ions, including lead, cadmium, mercury, and arsenic, through surface complexation and electrostatic interactions. These properties make LDHs promising materials for the treatment of industrial effluents, agricultural runoff, and contaminated groundwater. LDHs also play a crucial role in catalysis and photocatalysis. They can be used directly as heterogeneous catalysts or as precursors for the synthesis of mixed metal oxides with high catalytic activity. In photocatalysis, LDH-based materials and their composites have been extensively studied for the degradation of persistent organic pollutants under ultraviolet and visible irradiation.^32^ The lamellar structure promotes the homogeneous dispersion of active sites and improves charge separation, thus optimizing photocatalytic efficiency. Furthermore, coupling LDHs with semiconductor materials broadens their light absorption spectrum and enhances their redox performance. In the field of energy storage and conversion, LDHs have proven to be promising functional materials. Their large specific surface area, redox-active metal centers, and structural stability make them ideal candidates for supercapacitor and rechargeable battery electrodes. Furthermore, transition metal-based LDHs exhibit excellent electrocatalytic activity for key energy-related reactions, such as oxygen and hydrogen release, and water electrolysis, thus contributing to the development of sustainable and clean energy technologies.^33,34^ In fact, they have emerged as highly promising electrocatalysts for the oxygen evolution reaction (OER), owing to their compositional versatility, tunable structure, and facile synthesis. Several studies have demonstrated that their electrocatalytic performance can be significantly enhanced through rational design strategies; including morphology control, compositional tuning, and optimization of synthesis methods.^35–37^ Key factors such as electronic structure, active site exposure, and interlayer chemistry play a critical role in determining OER activity. Despite these advances, challenges related to catalytic efficiency and long-term stability remain, highlighting the need for further research toward practical applications. Despite advances in NiFe-LDH for seawater oxidation, their industrial application remains limited by challenges such as selectivity against the parasitic chlorine evolution reaction, the complex influence of ions and impurities, and long-term stability. Strategies such as doping, defect engineering, and the design of composites must be further explored to better understand reaction mechanisms and catalyst degradation. Finally, scaling up to industrial levels requires optimizing materials and operating conditions, as well as developing durable, cost-effective electrodes suited to real seawater conditions.

In biomedical applications, Layered double hydroxides (LDHs) such as MgAl-LDH are used due to their biocompatibility and ability to deliver drugs, genes, and biomolecules, improving bioavailability, controlled release, and anticancer therapy efficiency.^38,39^ Consequently, LDHs exhibit strong cellular penetration due to their positive charge, which enhances drug delivery efficiency and demonstrates their potential as carriers for genes, proteins, and imaging agents, highlighting their versatility. In addition to biomedicine, LDHs such as MgAl-LDH, ZnAl-LDH, NiAl-LDH, and MgFe-LDH are used as industrial materials including corrosion protection,^40,41^ barrier coatings,^42^ polymer nanocomposites,^43^ and flame-retardants where they enhance mechanical, thermal, and protective properties.^44–46^

Despite the remarkable performance of layered double hydroxides demonstrated at the laboratory scale, their transition toward industrial applications remains limited. Recent studies highlight that LDHs possess strong potential for large-scale deployment due to their low-cost precursors, structural tunability, and high efficiency in pollutant removal processes.^47^ However, several critical challenges must be addressed to enable their practical implementation. From a technological perspective, one of the main barriers is the stability of LDH materials under real operating conditions. Under industrial environments, LDHs are exposed to complex physicochemical conditions, including fluctuating pH, high ionic strength, and the presence of competing ions or organic matter. These conditions often lead to structural degradation, metal leaching, and loss of active sites, which significantly reduce their long-term performance and reusability.^48^ In addition, while many LDH-based systems exhibit excellent efficiency under controlled laboratory conditions, they frequently fail to maintain performance at high flow rates and large-scale continuous operations. This is particularly related to mass transfer limitations, particle aggregation, and poor mechanical stability, which hinder their integration into fixed-bed or membrane-based treatment systems.^49^ Another important limitation concerns the complexity of real wastewater matrices.^50^ Unlike synthetic solutions, industrial effluents (*e.g.*, from textile, tannery, or electroplating industries) contain a mixture of heavy metals, dyes, salts, and microorganisms. This complexity can result in competitive adsorption, surface fouling, and reduced selectivity of LDH materials, thereby limiting their efficiency in real applications.^51^ From an economic and commercial standpoint, challenges include the cost of large-scale synthesis, reproducibility of material properties, and regeneration efficiency. Although LDHs can be synthesized using low-cost or waste-derived precursors (*e.g.*, red mud, slag, or fly ash), scaling up these processes while maintaining consistent quality remains a significant challenge. Nevertheless, the use of industrial waste as a feedstock represents a promising strategy for reducing production costs and improving sustainability. Furthermore, the integration of LDH materials into existing wastewater treatment infrastructures remains underexplored. For industrial adoption, LDHs must be compatible with conventional technologies such as adsorption columns, catalytic reactors, or hybrid advanced oxidation processes. This requires the development of structured or composite materials (*e.g.*, LDH-coated supports, pellets, or membranes) with improved mechanical strength and operational stability. Overall, bridging the gap between laboratory research and industrial implementation requires a shift in focus from maximizing performance under ideal conditions to addressing durability, scalability, and process integration. Future research should prioritize pilot-scale studies, long-term stability assessments, and techno-economic analyses to accelerate the commercialization of LDH-based technologies for wastewater treatment.^52^

## 3. Structural and chemical properties of LDH materials

**Fig. 2:**
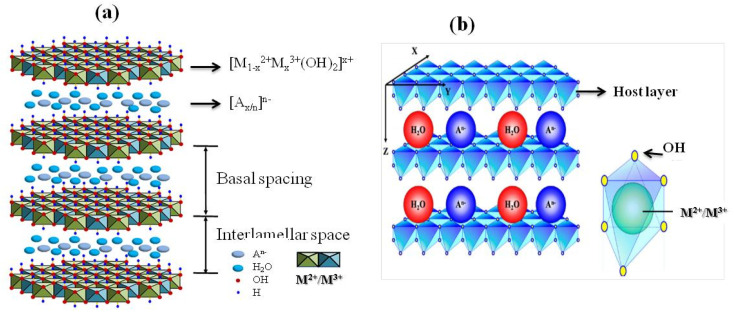
(a) Schematic representation of LDH structure adapted from ref. 53 with permission from MDPI, doi: https://10.3390/catal7090260, copyright 2017 (b) 2D LDH layered materials with a brucite-like structure, composed of M^2+^/M^3+^ cations, with interlayer anions and water molecules, adapted from ref. 54 with permission from MDPI, doi: https://10.3390/coatings10070669, copyright 2020.

Layered double hydroxides (LDHs) are two-dimensional inorganic materials with a brucite-like structure, composed of stacked hydroxide layers in which divalent (M^2+^) and trivalent (M^3+^) metal cations occupy octahedral sites coordinated by hydroxyl groups (Fig. 2).

**Fig. 3:**
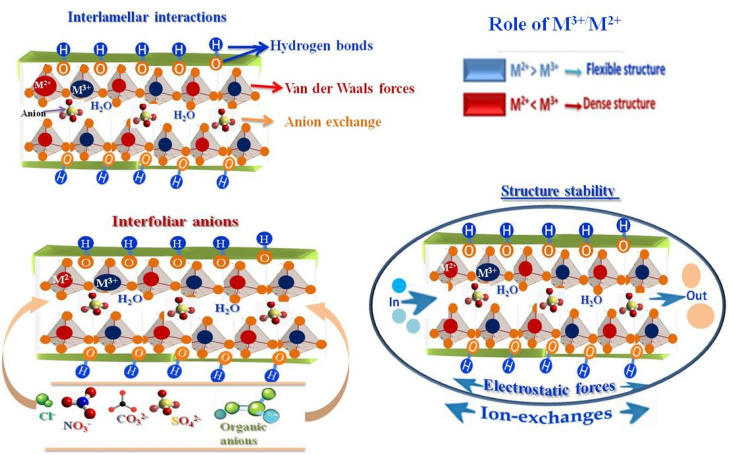
Structural and chemical features of LDH materials, highlighting interlayer interactions and the role of M^3+^/M^2+^ cations in lattice distortion, ion-exchange capacity, electrostatic forces, and interlayer anions.

The partial substitution of M^2+^ by M^3+^ generates a net positive charge on the layers, which is compensated by negatively charged interlayer anions and water molecules located in the interlamellar galleries. This layered architecture results in a well-defined lamellar structure, providing LDHs with high anion-exchange capacity, tunable chemical composition, and significant structural flexibility (Fig. 3). The ratio of M^2+^ to M^3+^ cations critically influences the charge density of the layers, the distortion of the octahedral sheets, and the interlayer spacing, thereby affecting lattice parameters, porosity, and overall structural stability.^26^ Interlayer anions, such as CO_3_^2−^, NO_3_^−^, Cl^−^, SO_4_^2−^, or organic anions,^55^ are held by electrostatic forces and hydrogen bonding with hydroxyl groups and water molecules, and they are exchangeable, enabling ion-exchange processes central to the chemical reactivity, adsorption, and functional versatility of LDHs. The nature, charge, and size of these interlayer anions strongly influence the physicochemical properties of LDHs.^56^ Highly charged anions, such as CO_3_^2−^, interact strongly with the hydroxide layers, enhancing structural stability and reducing interlayer spacing, whereas bulkier or monovalent anions lead to expanded interlayer galleries and increased flexibility.^26,55,56^ Structural defects and heterogeneity in the interlayer space further modulate charge distribution, active site accessibility, and the adsorption or catalytic performance of LDH materials, making them versatile for applications in ion exchange, catalysis, and environmental remediation.

**Fig. 4:**
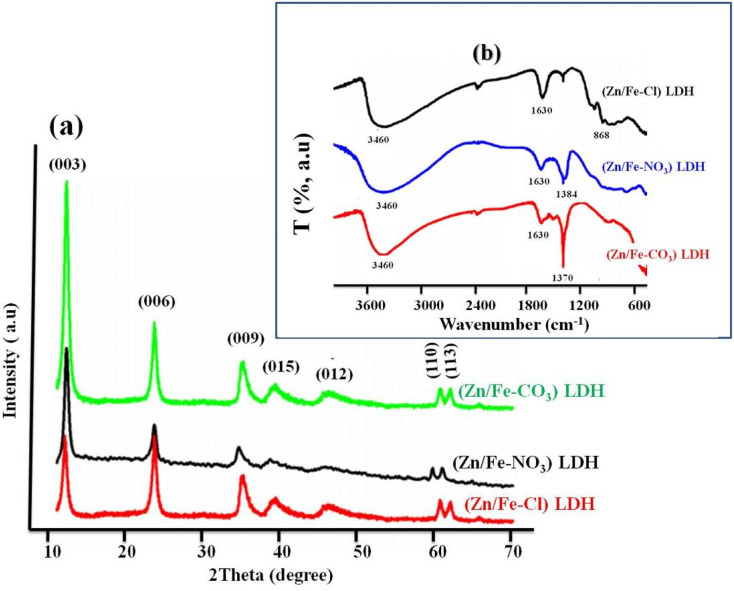
(a) Typical XRD patterns and (b) FTIR spectra of ZnFe-based LDHs with different interlayer anions (Cl^−^, NO_3_^−^ and CO_3_^2−^), adapted from ref. 58 with permission from Elsevier, doi: https://10.1016/j.cej.2011.10.070, copyright 2012.

As an illustrative example from ref. 57Fig. 4a shows the X-ray diffraction (XRD) patterns of the chloride ZnFe-LDH (ZnFe-Cl), Nitrate ZnFe-LDH (ZnFe-NO_3)_ and Carbonate ZnFe-LDH (ZnFe-CO_3_). All samples exhibit the characteristic diffraction peaks of layered double hydroxides, indexed along the (003), (006), (012), (015), (110), and (113) planes,^58^ thus confirming the formation of the LDH structure. A slight decrease in interplanar spacing is observed between ZnFe-Cl (7.9 Å), Zn/Fe-NO_3_ (7.89 Å), and ZnFe-CO_3_ (7.8 Å), which can be attributed to the higher charge density of carbonate anions.^57^ These results indicate that all synthesized ZnFe LDHs possess a well-ordered lamellar structure, regardless of the intercalated anion. The reduced basal spacing in carbonate-intercalated LDH reflects stronger electrostatic interactions between divalent CO_3_^2−^ anions and positively charged hydroxide layers, resulting in a more compact interlayer configuration than in LDH containing monovalent nitrate or chloride ions. Furthermore, FT-IR analysis confirms the structural integrity of the LDH network, as evidenced by similar M-O vibration bands around 1000 cm^−1^ and characteristic vibrations associated with hydroxyl groups at 1630 cm^−1^ and 3460 cm^−1^ for all samples Fig. 4b. Distinct absorption bands corresponding to interlayer anions were observed at approximately 1370 cm^−1^ for CO_3_^2−^, 1384 cm^−1^ for NO_3_^−^, and 868 cm^−1^ for Cl–O, thus confirming the successful and selective intercalation of the respective anions. These anion-dependent structural features are expected to influence the physicochemical properties and potential applications of ZnFe-LDH.

## 4. Tailored synthesis approaches for structural control

### 4.1. Synthesis

**Fig. 5:**
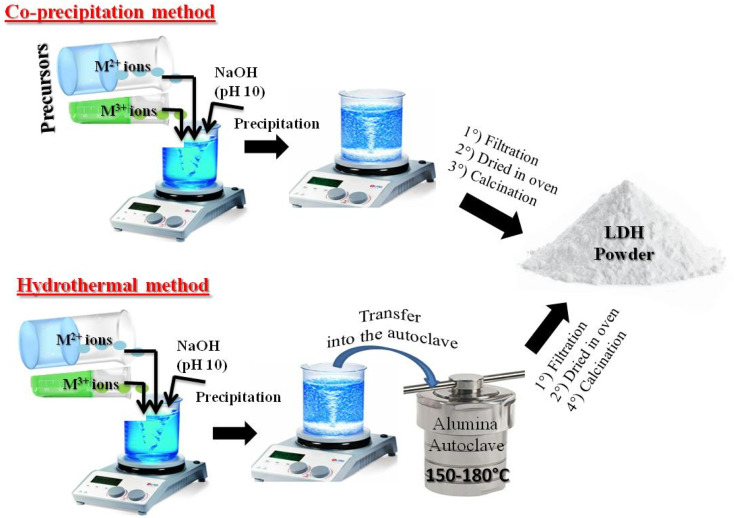
Typical schematic illustration of LDHs synthesis *via* co-precipitation and hydrothermal routes.

LDHs are most commonly synthesized using commercial metal salts (nitrates, chlorides, or sulfates of divalent and trivalent cations) because of their high purity, ready availability, and the straightforward control of reaction parameters. Among the various methods of material synthesis, coprecipitation and hydrothermal techniques are the most commonly used because of their simple, rapid and reproducible procedure, allowing homogeneous materials to be obtained, which easy control of composition (Fig. 5). The synthesis methods directly influence the chemical composition, crystallinity, morphology, layer size, nature of interlayer anions, and consequently, the physicochemical properties and application performance of the LDHs.

As shown in Table 1, hydrothermal, solvothermal, sol–gel, and microwave methods produce well-ordered sheets with high surface areas (100–300 m^2^ g^−1^) and variable crystallite sizes, while coprecipitation and mechanochemical routes give moderate crystallinity and smaller surface areas (70–150 m^2^ g^−1^). Electrochemical synthesis mainly forms uniform films with lower surface areas. The choice of method thus depends on the targeted crystallinity, surface area, and crystallite size, which govern performance in catalysis, adsorption, or other applications.

**Table 1:** Properties of (MgAl, ZnAl, NiAl, and MgFe)-LDHs according to the synthesis method

Method	Structural features	Interlamellar distance *d* (Å)	Crystallite size (nm)	Specific surface area (m^2^ g^−1^)	Ref.
Coprecipitation	Uniform lamellar layers, moderate crystallinity	7.6–8.0	10–25	80–150	59
Hydrothermal	High crystallinity, well-ordered sheets	7.8–8.2	20–40	100–200	32
Solvothermal	Palmate sheets, solvent-controlled morphology	7.7–8.3	15–35	120–220	37
Sol–gel	Homogeneous network, regular morphology	7.6–8.1	10–30	150–250	60
Microwave	Rapid nucleation, thin crystallites	7.8–8.0	5–20	180–300	61
Electrochemical	Films uniform LDH on substrate	7.6–8.0	15–30	50–120	62
Mechanochemical	Sheet formation, but low crystallinity	7.5–7.9	10–25	70–150	58

Controlled pH and addition rates during synthesis yield a well-ordered lamellar structure with evenly distributed cations, resulting in enhanced anion exchange capacity and structural stability of MgAl-LDH and ZnAl-LDH.^63^ Comparatively, hydrothermal synthesis, by involving heat treatment under pressure, significantly increases crystallinity and lamellar order, leading to structures where the basal planes exhibit stronger and sharper XRD peaks.^32,37^Table 2 summarizes the effects of hydrothermal operating parameters on the structure of the developed LDH materials. Furthermore, employing solvothermal methods with different solvents alters the morphology of the nanosheets and enhances accessibility to surface active sites, thereby improving the performance of LDHs in demanding applications like selective adsorption and photocatalysis. Meanwhile, alternative methods such as urea hydrolysis produce LDH with finer morphologies and high specific surface areas, well suited for pollutant adsorption and catalysis.^61^ Microwave-assisted synthesis, although less represented in global reviews, is known to accelerate nucleation and reduce crystallite size, leading to highly reactive nanostructures with large specific surface areas, which is particularly beneficial for electrochemical and catalytic applications,^21,64^ Finally, sol–gel or mechanochemical approaches affect cation dispersion and sheet size, thus influencing key properties such as specific surface area, thermal stability, or surface reactivity.^25^

**Table 2:** Effects of hydrothermal operational parameters on LDH structure

Operating parameter	Typical values	Structural effects	Ref.
Temperature	80–120 °C	Dominant nucleation, moderate crystallinity, thin sheets	65
	150–200 °C	High crystallinity, sheet growth, improved lamellar order	66
	>180–200 °C	Partial dehydroxylation, LDO formation	67
Autogenic pressure	1–5 MPa	Improved solubility and re-crystallization	68
Processing time	6–12 h	LDH with weak crystallization, small crystallite size	69
	24–48 h	Structural maturation, improved thermal stability	23
Initial pH	8–9	Partial formation, structural defects	70
	9–10	Well-crystallized LDH, stable structure	71
	>10.5	Precipitation of simple hydroxides	72
Solvent (solvothermal)	Water	Classic lamellar morphology	73
	Eau/Ethanol	Thinner nanosheets, surface ↑	51
	Polyols	Advanced morphological control	62
Precursor concentration	0.05–0.1 M	Homogeneous LDH, low aggregation	74
	>0.3 M	Aggregation, structural defects	57

However, the synthesis method plays a crucial role in determining the structural and physicochemical properties of layered double hydroxides (LDHs), which ultimately influence their performance in different applications. For instance, co-precipitation, one of the most commonly used synthesis routes, typically produces LDHs such as MgAl-LDH or ZnAl-LDH with relatively small crystallite sizes and high surface areas. These characteristics are particularly advantageous for applications in adsorption and water treatment, where a high density of active sites is required. In contrast, hydrothermal synthesis often leads to LDHs with improved crystallinity and more ordered layered structures. LDHs such as NiFe-LDH or CoAl-LDH prepared through hydrothermal treatment have demonstrated enhanced electrochemical and catalytic properties, making them promising materials for electrocatalysis and energy-related applications. The increased crystallinity and structural stability obtained by this method can improve electron transfer and catalytic efficiency. Similarly, sol–gel and reconstruction (memory effect) methods allow better control over particle morphology and interlayer composition. These approaches are particularly useful when designing LDHs for drug delivery, controlled release systems, or functional nanocomposites, where precise control of particle size and interlayer chemistry is essential. Overall, recent studies highlight that optimizing the synthesis strategy is essential to tailor LDH properties for specific applications. Rather than considering applications independently, it is important to understand how composition (*e.g.*, MgAl-LDH, ZnAl-LDH, NiFe-LDH), morphology, crystallinity, and interlayer chemistry are directly influenced by the synthesis method and determine the resulting performance. Table 3 summarizes these aspects to illustrate the relationship between the synthesis method and the structure of LDH materials, as well as their main applications.

**Table 3:** Relationship between synthesis method, LDH composition, and main applications^75–79^

Synthesis method	LDH composition	Structural characteristics	Main applications
Co-precipitation	MgAl-LDH, ZnAl-LDH	Small crystallites, high surface area	Adsorption, water treatment
Hydrothermal	NiFe-LDH, CoAl-LDH	High crystallinity, ordered layers	Electrocatalysis (OER), energy conversion
Sol–gel	MgFe-LDH	Controlled morphology	Catalysis
Reconstruction	Various LDHs	Rehydration ability, tunable interlayer chemistry	Drug delivery, functional materials

Conventional LDH synthesis methods such as co-precipitation and hydrothermal routes are well established, robust, and reproducible, but they often rely on high-purity reagents and controlled conditions, which may limit their sustainability and large-scale applicability. Recently, increasing attention has been given to the synthesis of LDHs from waste-derived precursors as a more sustainable alternative. Although promising in terms of cost reduction and environmental impact, these approaches still face challenges related to variability of raw materials and control over final material properties, requiring further optimization. In this context, natural resources and industrial wastes have emerged as promising substitutes for conventional chemical reagents.^80^ These materials, including mineral ores, brines, red mud, slag, or other industrial by-products, contain significant amounts of metal ions that can be recovered and utilized for LDH synthesis. The use of such resources not only reduces the overall cost of production but also contributes to waste valorization and environmental protection. Therefore, the synthesis of LDHs from natural and waste-derived precursors has attracted increasing attention, particularly in studies focused on environmental applications, as it aligns with the principles of sustainable chemistry and circular economy. The synthesis of LDH from clays, metallurgical waste or industrial by-products constitutes a sustainable and valuable approach, making it possible to produce functional lamellar materials while reducing the environmental footprint.^81,82^ Extraction and activation of natural precursors by dissolution, calcination, or hydrothermal processes promotes the formation of crystalline LDH with typical specific surface areas of 50–150 m^2^ g^−1^ and crystallites of 10–30 nm.^83,84^ These studies have shown that synthesizing LDHs from natural resources or waste is not only feasible but also produces functional materials with performance comparable to LDHs developed from commercial reagents, while providing notable environmental and economic advantages.

### 4.2. Impact of chemical composition on structural properties

The chemical composition of LDHs significantly influences their structural properties, from crystal organization to specific surface area and thermal stability. Classical binary LDH systems such as MgAl-LDH, ZnAl-LDH, NiAl-LDH, and FeAl-LDH are among the most studied due to their well-defined brucite-derived layered structures, with lattice parameters and interlayer spacings determined by the specific divalent and trivalent cations. MgAl-LDH and ZnAl-LDH exhibit high crystallinity and structural stability, while NiAl-LDH and FeAl-LDH systems display increased octahedral distortion due to the ionic sizes and electronic structures of the transition cations.^17^ Incorporating ternary or multicationic systems enables precise tuning of sheet charge density, the distribution of active sites, and textural characteristics. These systems generally exhibit increased structural heterogeneity, which can induce controlled lamellar disorder favorable to catalytic and adsorption applications.^85^ Isomorphic substitution and metal doping are effective strategies for locally modifying the electronic structure and geometry of the layers without altering the overall lamellar architecture. Incorporating Fe^3+^, Co^2+^, or La^3+^ cations can introduce structural defects and alter surface reactivity.^86^ Moreover, the type of interlayer anions, whether inorganic (Cl^−^, NO_3_^−^, CO_3_^2−^, SO_4_^2−^) and organic or polyatomic, significantly affects structural stability and layer arrangement, while also influencing the accessible surface area and anion exchange capacity.^25,87^Table 4 summarizes the effects of dopants, as well as added cations and anions, on LDH-materials. It provides a concise view of how the type of substitution influences the structure and performance of LDHs in environmental applications.

**Table 4:** Impact of substitutions on LDH composition, structure, and properties

Composition/parameter	Nature of doping/cation added	Structural effects observed	Physico-chemical properties	Thermal stability	Ref.
Binary systems: MgAl-LDH	No specific doping agents, main cations	–High crystallinity for MgAl-LDH, and ZnAl-LDH.	Specific surface area 50–200 m^2^ g^−1^, moderate porosity, high anion exchange capacity	−400–500 °C for MgAl-LDH, ZnAl-LDH.	88
ZnAl-LDH		–Octahedral distorsion for NiAl-LDH et FeAl-LDH.		−350–450 °C for (Ni–Al, Fe–Al)	
NiAl-LDH					
Fe-Al-LDH					
Ternary/multi-cationic systems	Additional cations: Zn^2+^, Mg^2+^, Co^2+^, Ni^2+^, Fe^3+^, Al^3+^	Controlled lamellar heterogeneity, modulation of active sites, partial disorder	Specific surface area 70–250 m^2^ g^−1^, increased porosity, improved catalytic dispersion	350–500 °C depending on the composition	89
Isomorphic substitution/metal doping	Common dopants: Fe^3+^, Co^2+^, La^3+^, Mn^2+^, Cr^3+^	Modification of parameters, introduction of defects, variation in surface reactivity	Increase or decrease in specific surface area depending on the dopant, modification of the basicity/acidity of the sites, adjustable chemical stability	350–500 °C depending on the doping	86
Incorporation of functional anions	Interlayer anions: CO_3_^2−^, NO_3_^−^, Cl^−^, SO_4_^2−^, organic anions	Variation in interlayer spacing, modulated interlayer interactions	Adjustable specific surface area, modified anion exchange capacity, structural flexibility	300–450 °C for organic anions, >400 °C for inorganic anions	87
Effect of the M^2+^/M^3+^ ratio	Variation of the ratio Mg^2+^/Al^3+^, Zn^2+^/Al^3+^, Ni^2+^/Al^3+^	Optimization of charge density, balance between structural stability and accessible surface area	Influence on porosity, specific surface area and reactivity, maximum adsorption capacity for intermediate ratios	350–500 °C depending on the ratio chosen	17

### 4.3. Effect of calcination: LDH → LDO

Calcination of LDHs converts them into LDOs by dehydroxylation and decarbonation, removing interlayer ions and partially collapsing the sheets to form amorphous or crystalline phases depending on temperature.^90^ Calcination temperature plays a key role: moderate temperatures (400–500 °C) produce reactive, amorphous LDOs, whereas higher temperatures promote crystallization of mixed oxides and secondary phases, affecting cation composition and catalytic performance.^17^ The ‘memory effect’ enables LDOs to rehydrate and partially restore their original LDH structure, providing reversible behavior and maintaining anion exchange capability.^91^ This conversion impacts textural characteristics, as pore formation and higher surface area improve chemical activity and efficiency in adsorption and heterogeneous catalysis. Compared to LDH, LDOs offer greater surface area and porosity but with some loss of crystallinity. This combination of properties endows LDH-derived materials with remarkable versatility for environmental and catalytic applications.^17^Table 5 shows the effects of various treatments on the structural, textural, and thermal properties of the LDH. It highlights how modifications such as calcination, substitution, or post-synthesis treatments can alter the layered structure, enhance porosity, and improve thermal stability, providing insights into the material's suitability for adsorption, catalysis, and other functional applications.

**Table 5:** Effects of experimental parameters on the structure, porosity, and thermal stability of LDHs.^17,90^

LDH type	Operating parameter	Typical values	Structural effects
Carbonated MgAl-LDH	Thermal treatment	400–500 °C	Reactive amorphous LDO, partially preserved lamellar layers, strongly bound CO_3_^2−^ anions maintain local order
		>600 °C	Formation of MgAl_2_O_4_ spinel, partial loss of lamellar order
	Dehydroxylation/Decarbonation	Controlled thermal process	Collapse of the layers, release of intercalated OH^−^ and CO_3_^2−^
	Memory effect	Rehydration	Partial reconstruction of the lamellar layers, recovery of anion exchange
	Specific surface area	50–150 m^2^ g^−1^	Creation of pores and active sites, adsorption and catalytic reactivity
Sulphated MgAl-LDH	Thermal treatment	400–500 °C	Amorphous LDO, partially retained leaflets
		>600 °C	Partial formation of MgAl_2_O_4_ spinel, lamellar disorder
	Dehydroxylation/decarbonation	Thermal process	Lamellar collapse and release of OH^−^ and SO_4_^2-^
	Memory effect	Rehydration	Partial reconstruction of lamellar layers, capacity for anion exchange
	Specific surface area	50–160 m^2^ g^−1^	Increased surface area and porosity, improved catalytic activity
Carbonated ZnAl-LDH	Thermal treatment	400–500 °C	Amorphous LDO, partial retention of leaflets
		>600 °C	Formation of ZnAl_2_O_4_ spinel, loss of lamellar order
	Dehydroxylation/decarbonation	Thermal process	Collapse of the layers and release of intercalated CO_3_^2−^
	Memory effect	Rehydration	Partial restoration of the lamellar layers
	Specific surface area	60–180 m^2^ g^−1^	Increased surface area and porosity, catalytic
Carbonated FeAl-LDH	Thermal treatment	400–500 °C	Amorphous LDO with partial retention of the lamellar structure
		>600 °C	Formation of Fe_2_O_3_ and FeAl_2_O_4_ spinel, lamellar disorder
	Dehydroxylation/decarbonation	Thermal process	Lamellar collapse and release of intercalated anions
	Memory effect	Rehydration	Partial reconstruction of the structure, recovery of anion exchange
	Specific surface area	60–170 m^2^ g^−1^	Pore creation and increased surface area, improved adsorption and catalytic activity
Sulphated CoAl-LDH	Thermal treatment	400–500 °C	Amorphous LDO with partially preserved sheets
		>600 °C	Crystallization in CoAl_2_O_4_ spinel, loss of lamellar order
	Dehydroxylation/decarbonation	Thermal process	Sofa collapse, OH^−^ and SO_4_^2−^ release
	«Memory effect»	Rehydration	Partial restoration of the lamellar structure
	Specific surface area	60–180 m^2^ g^−1^	Pore creation and surface area increase, catalytic

## 5. Environmental applications of LDHs

### 5.1. Adsorptive behavior of LDHs

Layered double hydroxides (LDHs) are characterized by their layered structure, high specific surface area, and anion exchange capacity, making them excellent candidates for environmental applications. Their chemical versatility allows tuning of the metal composition (Mg, Al, Zn, Fe, Co, *etc.*) and the intercalated anions (CO_3_^2−^, SO_4_^2−^, NO_3_^−^), enabling optimized selective adsorption and catalytic activity. They are particularly effective for adsorbing heavy metals (Pb^2+^, Cd^2+^, Cu^2+^, or Ni^2+^) due to the combination of ionic interactions, surface complexes, and ion exchange.^9,18^ The adsorption capacity of LDHs is strongly influenced by the nature of the M^2+^/M^3+^ cations and the solution pH, reaching saturation levels of several tens of mg g^−1^ depending on the metal type and the material's specific surface area. Similarly, LDHs can remove toxic anions such as F^−^, NO_3_^−^, PO_4_^3−^, and Cr(vi) *via* anionic substitution and interlamellar trapping mechanisms, while maintaining high chemical stability under various aqueous conditions.^92^ LDHs also exhibit excellent performance for adsorbing dyes and organic pollutants, including azo-dyes, pesticides, and pharmaceuticals.^93,94^ The combination of physical adsorption on the surface and intercalation within the layers allows for efficient trapping of organic molecules, sometimes enhanced by post-synthesis treatments (calcined LDH or composites with oxides). LDHs and their composites are effective for adsorbing metals, anions, dyes, and pharmaceutical pollutants, with capacities ranging from 20 to 1300 mg g^−1^ depending on the material and pollutant (Table 6). The experiments are performed in batch mode over varying pH and time ranges.

**Table 6:** Adsorption capacities and mechanisms of LDHs and their composites for various pollutants in water

LDH	Pollutant	Key conditions	Capacity *q*_max_ (mg g^−1^)	Adsorption mechanism	Ref.
MgLa-LDH	PO_4_^3−^	Batch, pH 6–7, contact 25–60 min, 25 °C	87.23	Chemisorption monolayer, ion- exchange, surface complexation	91
MgAl-LDH@Biochar	Cu^2+^, Co^2+^, Pb^2+^, PO_4_^3−^	pH 5–7, 30–120 min, dose 0.5–1 g L^−1^	Cu^2+^: 25.8; Co^2+^: 15.0; Pb^2+^: 40.4; PO_4_^3−^: 21.8	Ion-exchange + surface complexation	89
FeMg-LDH@ Bentonite	Pb^2+^, Cd^2+^	Batch, pH 6, 2 h, 2 g L^−1^	Pb^2+^: 1397.6; Cd^2+^: 510.2	Surface complexation + ion exchange + precipitation	95
Activated carbon@ MgAl-LDH (composite)	PO_4_^3−^	Batch, pH 6, 22 °C, 1 h	209.8–337.2	Electrostatic + ion-exchange + inner-sphere complexation	96
(CO_3_/Cl) MgZnAl-LDH	Textile dye IDB	pH 4.6, 6 h, dose 2 g L^−1^	94.5	Chemisorption	97
MgAl-LDH	Reactive Black 5	30 °C, pH 5–6, 4 h	61.3	Anion exchange + surface adsorption	68
MgAl-LDH	Methylene blue, amoxicillin	pH 11, 50 mg L^−1^ adsorbent, 120 min	114.94	H-bonding + Van der Waals	93
			48.08		
Cellulose@CoFe LDH composite	Sulfamethoxazole cefixime	pH 5, 0.1 g adsorbent	272.13	Surface Binding, complexation	98
			208.00		
MgFe-LDH@ Biochar	Tetracycline, ciprofloxacin	Batch, wide pH range	77.04	Complexation + H-bonds	99
		Batch, wide pH range	66.74		
Activated carbon@ MgFe-LDH composite	Phenol	—	138.69	Chemical bonding + π–π interactions	100
Carbon-LDH materials	Methylene blue	*T*∼30 °C, pH 6.8	328.95 (MO); ∼80–122 (MB)	Langmuir adsorption	101
	Methyl orange, congo red, crystal violet	Varied	100–400	Electrostatic+ π–π + H-bonding	102

**Fig. 6:**
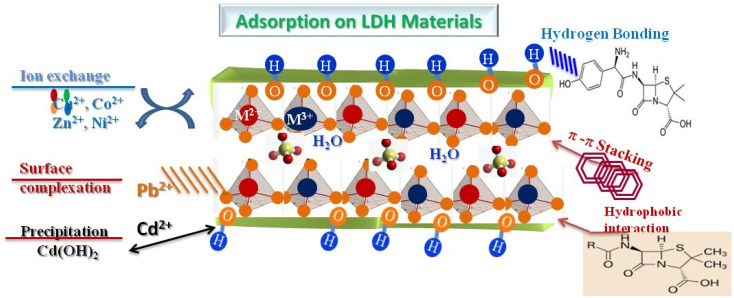
Adsorption mechanisms of contaminant on LDH materials.

The primary mechanism is ion exchange, where interlayer anions of LDH, such as CO_3_^2−^, NO_3_^−^, or Cl^−^, are replaced by anions present in the solution (Fig. 6). This interaction is particularly effective for capturing heavy metals and organic anions (dyes, pharmaceuticals). The efficiency of the exchange is highly dependent on pH, the nature and charge of the anion, and the availability of interlayer sites within the lamellar structure. Simultaneously, surface adsorption occurs *via* electrostatic interactions. Hydroxyl groups in LDH layers can interact with metal cations through chemical complexation, or with polar organic molecules through hydrogen bonding and electrostatic interactions. For Pb^2+^, Cd^2+^ or Cu^2+^ ions, they can bind directly to hydroxyl sites, while negatively charged organic molecules are attracted to the positively charged surfaces of LDH. Certain molecules or ions can also be trapped in the interlayer spaces. This intercalation mechanism is often observed for organic or neutral anions and allows for partial confinement of contaminants, thus increasing adsorption capacity. The size and polarity of the contaminant, as well as the expansion of the interlayer space, strongly influence this type of adsorption. LDHs can be combined with other materials to form composites, for example with carbon (LDH@C) or semiconducting oxides (LDH@TiO_2_, LDH@ZnO), which introduces synergistic mechanisms. In LDH@C composites, π–π and electrostatic interactions enhance the adsorption of aromatic molecules, while LDH@oxide enables the photodegradation of organic pollutants while maintaining complementary adsorption. Finally, several factors influence the adsorption performance on LDH: the pH of the solution, which determines the surface charge and the ionic species of the contaminant; the temperature, which affects the kinetics and equilibrium of adsorption; the LDH dosage, which conditions the maximum capacity; and the structural composition of LDH, including the M^2+^/M^3+^ cation ratio, the nature of the interlayer anions and the specific surface area. In summary, contaminant adsorption on LDHs results from a combination of ion exchange, surface adsorption, intercalation, and, in the case of composites, synergistic mechanisms, making these materials particularly effective for treating a wide range of inorganic and organic pollutants. Understanding and modulating these mechanisms is essential for optimizing the environmental performance of LDHs.

### 5.2. Advanced applications of LDH catalysts and their composites in heterogeneous catalysis: focus on the degradation of drug residues

Heterogeneous catalysis emerges as a promising approach for the removal of various organic pollutants. Among emerging catalytic materials, LDHs and their composites stand out due to their modular structure, high adsorption capacity, and catalytic versatility. These materials are particularly effective in treating pharmaceutical residues in wastewater. In advanced oxidation processes, LDHs are used as catalysts or co-catalysts to activate powerful oxidants such as hydrogen peroxide (H_2_O_2_), persulfate (PDS), or peroxymonosulfate (PMS), generating highly reactive radical species (˙OH, SO_4_˙^−^). These species are capable of attaching to and breaking down the aromatic and functional structures of pharmaceutical residues, leading to their mineralization into largely harmless products (CO_2_, H_2_O).^103^ For example, LDHs containing transition metals (Fe, Co, Cu, *etc.*) significantly improve PMS activation and radical generation for the degradation of pharmaceutical pollutants, with degradation efficiencies often exceeding 90%.^104^ LDH composites, such as NiFe-LDH@AC, can enhance catalytic performance by efficiently activating persulfate to generate reactive sulfate radicals, enabling the heterogeneous degradation of pollutants like the antibiotic norfloxacin.^105^ This synergy arises from increased active surface area, facilitated electron transfer, and catalyst stabilization.^106^ Thermal treatment of LDHs produces mixed oxides (LDOs) with structural defects and extra active sites, enhancing their reactivity in PAOs. These LDOs typically show superior catalytic activity and recyclability compared to unmodified LDHs.^103^Table 7 summarizes the main steps involved in the adsorption or degradation of pollutants by LDH-materials, highlighting the mechanistic aspects of each process and the specific role of the LDH material in enhancing performance. However, despite these advances, challenges remain, including a thorough understanding of the reaction mechanisms, minimizing metal leaching, and optimizing for large-scale application in real-world water treatment.^107^ Consequently, LDHs and their composites provide a robust and versatile catalytic platform for the degradation of pharmaceutical residues *via* advanced heterogeneous processes, including adsorption, oxidant activation, and radical generation. Their modularity allows for targeted design to optimize efficiency under real-world environmental conditions, making them ideal candidates for next-generation water purification technologies.

**Table 7:** Key steps of the photocatalytic process and associated mechanisms, highlighting the specific role of LDH materials^102,104,106^

Step	Key process	Mechanistic description	Specific role of LDH
1	Light absorption	Under UV or visible irradiation, LDH or the LDH/semiconductor composite absorbs photons and generates electron–hole pairs (e^−^/h^+^)	Bandgap adjustment by doping or heterojunction
2	Generation of charges	Excitation of electrons from the valence band to the conduction band	Lamellar structure facilitating charge migration
3	Separation of charges	Directional transfer of electrons and holes at the LDH/semiconductor interface, limiting their recombination	Formation of efficient heterojunctions
4	Pollutant adsorption	Adsorption of pharmaceutical molecules on the surface or within the interlayer space	Positively charged leaves and high adsorption capacity
5	Oxygen reduction	Electrons react with O_2_ to form superoxide radicals (˙O_2_^−^)	Redox active sites (Fe, co, Ni, Cu…)
6	Oxidation of water/OH^−^	The holes oxidize H_2_O or OH^−^ to generate hydroxyl radicals (˙OH)	Hydroxylated surface rich in –OH groups
7	ROS generation	Formation of highly reactive ROS (˙OH, ˙O_2_^−^, ^1^O_2_)	Stabilization and amplification of reactive species
8	Radical attack	ROS attack the aromatic and functional bonds of pollutants	Proximity between surface and pollutant is favored by adsorption
9	Mineralization	Final oxidation to CO_2_, H_2_O and inorganic ions	Maintenance of catalytic activity and recyclability

**Fig. 7:**
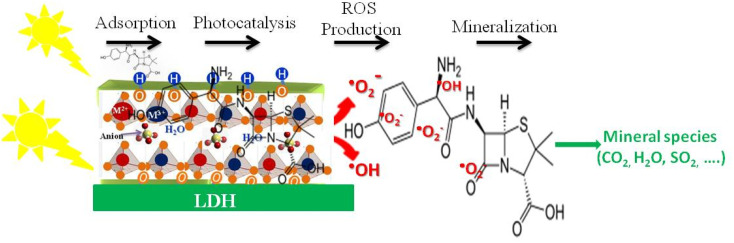
Schematic representation of the photocatalytic degradation mechanism of organic pollutants on LDHs under light irradiation.

Under UV or visible light, LDHs or LDH-semiconductor composites absorb photons to generate electron–hole pairs, initiating the photocatalytic process. The lamellar structure of LDHs facilitates charge separation and migration to the surface, where redox reactions occur. In pure LDHs, rapid electron–hole recombination can limit efficiency, but in composites, heterojunctions promote directional charge transfer and extend the lifetime of active charges. Photoexcited electrons reduce oxygen to superoxide radicals, while holes oxidize water or hydroxyl ions to hydroxyl radicals. These reactive species degrade pollutants, and the adsorption of contaminants on the LDH surface enhances their interaction with radicals, improving overall photocatalytic performance. The degradation of drug residues then occurs through a series of non-selective radical reactions. Hydroxyl and superoxide radicals attack the most reactive bonds of polluting molecules, such as aromatic rings, amine groups, or alkyl side chains. This attack leads to the progressive disruption of the molecular structure, the formation of lower molar mass intermediates, and their subsequent oxidation until partial or total mineralization into carbon dioxide, water, and inorganic ions. In certain doped or modified LDH systems, the presence of redox-active metal cations can also contribute to additional electron transfers, enhancing the generation of oxidizing species and accelerating the degradation kinetics. Thus, the photodegradation mechanism of pollutants by LDH relies on a synergy between adsorption, photo-induced excitation, efficient charge separation, and the production of reactive oxygen species. This combination gives LDHs and their composites a particular effectiveness for the degradation of persistent pharmaceutical pollutants, while offering great design flexibility for optimizing activity under visible light and realistic environmental conditions. Fig. 7 illustrates the generation of photo-excited electron–hole pairs upon light absorption by the LDH material, the subsequent formation of reactive oxygen species (ROS) such as ˙OH and ˙O_2_^−^, and their role in oxidizing adsorbed organic pollutants. The LDH structure facilitates charge separation and provides active sites for adsorption, enhancing the overall photocatalytic efficiency.

Table 8 summarizes the type of catalyst, the target pollutant, the optimized operating conditions and the degradation efficiency or removal rate along with the reaction time reported in the literature. LDHs and their composites have proven effective for the degradation of organic pollutants such as pharmaceuticals and dyes under UV or visible light irradiation. Carbonated ZnCr-LDH or modified LDHs (carbon doped ZnCr-LDH) have shown high degradation rates (77–90%) in relatively short times, thanks to improved light absorption, efficient separation of photoexcited charges, and enhanced adsorption of pollutants on the catalyst surface.^108^ Composites incorporating semiconductors, conductive supports (g-C_3_N_4_, MXene, h-BN), or S-scheme heterojunctions promote electron–hole separation, extending the lifetime of active charges and increasing the production of reactive radicals responsible for pollutant degradation. These advances not only allow the efficient treatment of pharmaceutical residues such as ibuprofen, ciprofloxacin, tetracycline, gemifloxacin and oxytétracycline, but also extend the application of LDHs to other emerging contaminants. The performance of LDHs strongly depends on their metal composition, lamellar structure, the presence of dopants or conductive supports, and their adsorption capacity, making these materials a versatile and promising catalytic platform for the remediation of contaminated water.

**Table 8:** Overview of research on the photocatalytic degradation of pollutants using Layered Double Hydroxides (LDHs) and their composites

Catalyst	Pollutant^a^	Optimized operating conditions	Degradation/time	Ref.
g-C_3_N_4_@ZnFe-LDH	TC	UV light + oxone (peroxymonosulfate) to generate SO_4_˙^−^ and ˙OH, combined with LDH composite	92.4% in 30 min	109
Zn-Co-LDH@ Biochar	GEM	UV light irradiation, catalyst loading optimized	∼92.7% in 200 min	110
LDH@Fe_3_O_4_-Ag	TC	Irradiation visible, 90 min, pH 9, initial TC 35 ppm, photocatalyst 0.05 g L^−1^	96.4% in 200 min	111
Zn-TMU-5@30%Ni-Ti -LDH	SUL	Natural solar light, 0.1 g L^−1^ catalyst	>98% in 45 min	112
g- C_3_N_4/_@ZnFe-LDH	TC	UV + oxone (persulfate source) (US assist)	100% TC in 25 min	113
g-C_3_N_4_/MgZnAl-LDO	TC	Visible light	100% in 120 min)	114
La-NiAl-LDH@hematene composite	RIF	Visible light, charge separation improved	≈ 89.2% in 120 min	94
RP@ZnAl-LDH	TC	Visible light	89% in 90 min	91
MgAl-LDH@g- C_3_N_4_@Ag_3_PO_45_	MB	Visible light (150 W mercury lamp), pH = 11, catalyst 0.05 g/100 mL, dark adsorption step, 45 min irradiation	99% in 45 min	115
O-doped g- C_3_N_4_@LDH	MB	Visible light, catalyst dose & pH increased to maximize ^˙^OH radicals	≈98.5%	96
ZnAl-LDH@POM	MB	Visible light irradiation, catalyst loading 0.6 g L^−1^, [MB] ∼7.5 mg L^−1^, pH ∼6	∼78% (varies with composition)	116

a Tetracycline (TC), gemifloxacin (GEM), sulfamethoxazole (SUL), oxytétracycline (OXY), ibuprofen (IBU), rifampicine (RIF), methylene blue (MB). Red phosphorus (RP).

The thermal stability of LDHs remains a critical parameter for their reuse as adsorbents or catalysts. Calcination, for example at 600 °C, can sometimes lead to the formation of LDOs, resulting in the loss of lamellar structure and porosity, which reduces their efficiency. To address this issue, it is preferable to use LDHs as they are while enhancing their thermal stability, allowing their reuse by burning residual molecules and/or by-products at 600 °C without altering the structure or surface. Strategies such as the careful selection of cations, metal doping, and the integration of stable supports help reinforce thermal robustness. Thermally stable LDHs can thus be efficiently recycled, maintaining their performance and enabling the safe destruction of by-products, thereby optimizing their role in environmental applications.

Overall, the above discussion highlights that while LDH-based materials demonstrate remarkable environmental performance and significant application potential, addressing the remaining challenges is essential to fully bridge the gap between laboratory research and real-world industrial implementation. These considerations provide a solid basis for the general conclusions of this review. Layered double hydroxides are highly promising materials for environmental applications due to their tunable composition, high surface area, and strong anion-exchange capacity, which enable efficient adsorption and photocatalytic degradation of pollutants. After suitable modification or coupling with semiconductors, they exhibit enhanced visible-light activity, improved charge separation, and higher generation of reactive species, making them effective for water and air purification. Their low cost, structural flexibility, and easy synthesis further strengthen their application potential. However, challenges such as scalability of synthesis, structural stability, particle recovery, and cost of some doped systems still limit their industrial deployment. Overall, LDHs remain strong candidates for environmental remediation, provided that efforts focus on improving large-scale production, durability, and practical implementation.

## 6. Conclusion

Layered double hydroxides (LDHs) and their composites represent a multifunctional class of materials with significant potential for the removal of organic pollutants from water. Their unique lamellar structure, compositional versatility, and chemical stability allow for effective tuning of physicochemical properties such as specific surface area, porosity, and adsorption capacity, which are critical for heterogeneous catalysis and photocatalysis applications. Combining LDHs with semiconductors, conductive supports, or dopants enhances the separation of photoexcited charges and promotes the generation of highly reactive radicals, resulting in high degradation rates for a wide range of pollutants, from dyes and pharmaceutical residues to emerging contaminants such as endocrine disruptors and microplastics. Analysis of the literature shows that the efficiency of LDHs strongly depends on their metal composition, morphology, post-synthesis treatments, and their ability to interact with pollutants through adsorption and oxidant activation. These characteristics make LDHs and their composites a versatile and promising catalytic platform for advanced water treatment technologies. Future perspectives include optimizing LDH-based composites for synergistic activation of multiple oxidants, extending visible-light responsiveness, and valorizing materials from natural resources or waste, paving the way toward effective and sustainable water remediation solutions.

## Author contributions

Z. Bouziane: conceptualization, literature search/data curation, writing – original draft, F. Amor: visualization, investigation; formal analysis, S. Fatine: methodology, writing – original draft preparation, J-M. Nunzi: review & editing, A. Laghzizil, writing – review & editing.

## Conflicts of interest

There are no conflicts to declare.

## Data Availability

No primary research results, software or code have been included and no new data were generated or analysed as part of this review article.
